# Supplementary material for: The experience of maternal mental distress in The Gambia: A qualitative study identifying idioms of distress, perceptions of contributing factors and the supporting role of existing cultural practices
Source: PLOS Glob Public Health. 2023 Sep 7;3(9):e0002329. doi: 10.1371/journal.pgph.0002329 (PMC10484451; doi:10.1371/journal.pgph.0002329)
Supplement: S1 Text — (DOCX) [file pgph.0002329.s002.docx]

**S1 Text**

**Focus Group Discussion Guide by Informant Group**

**Pregnant Women**

1. *Do women sometimes experience mental health issues during pregnancy?*

*Ndakh gigen yii dang deh lego-leag ham hel bu dalut (si missal, nakhare, jahleh, ak ragal) su nyu biree?*

1. What are the possible effects of mental health issues during and after pregnancy? How severe can it be? (For the mother and her baby)?

*Lan moi jurel lu hel bu dalut si gigen bu birr ak su wosine? Nakala buna garawee? (si yaai bi ak doomam)*

1. What kinds of things do women typically do to help themselves if they experience mental health issues?

*Yan mbirr la gigen yii warr di deff purr ndimbaleh sen bopa su nyu hameh jahleh, nakhare, walla hel bu dalut?*

1. Who can a pregnant woman go to for help or support when she feels anxious or depressed?

*Si kanla gigen bu birr muna dem purr ham ndimbal su ameh yeek-yeek ngi jahleh walla hel bu dalut?*

1. What do you think might help women deal with stress, anxiety, and depression during pregnancy?

*Lan ngen fox ne dina ndimbaleh gegen yii si sen wallu nakhare, jahleh ak hel bu dalut sun nyu birree?*

1. Do you think singing together would be beneficial for women who are experiencing stress, anxiety, or depression during and after pregnancy? If so, why? If not, why not?

*Ndakh fox ngen neh woi mbolo dina ham njerign si gigen yii ham nakhare, jahleh, walla helbu dalut su nyu birree ak su nyu wosine? Su hameh, lan mo takh? Walla su hamute lan mo ko waral?*

1. What kind of singing or music do you think would be beneficial (if any)?

*Yan fasong ngi woi walla misik ngen fox ne di ham njerign? (su ameh)?*

**Kanyeleng**

1. What do you call your group?

*Lan gen deh yoyeh sen mbotai bi?*

1. What kinds of activities does your group engage in?

*Yan fasong ngi yangu-yangu la sen mbotai bi di yangu?*

1. Can music help people deal with their stress, worries, and concerns? If so, how?

*Ndakh misik muna dembaleh nengi si sen hol bu nakhare, jahleh, ak buga-buga yii?Su deh neka na, nakala?*

1. What kinds of support do you give to people experiencing sadness or mental distress?

*Yan fasong ngi ndimbal ngai jokh nengi nyu nga ham neh nyngi ham hol bu nakhre, walla hel bu dalut?*

1. What kinds of songs are sung to babies? Follow up: Can you sing any of these songs?

*Yan woi lanyo woyal lirryi? Ndakh munnga woi bena si woyi?*

- 1. Who knows these songs and who sings them to babies?

*Kan mo kham woyi ak kan moi ko woyal lirryi?*

- 1. What is the benefit of these songs/ why are these songs performed?

*Lan moi njerign woi/ lan motakh nyun koi deff?*

1. What kinds of songs and dances are performed at naming ceremonies?

*Yan fasong ngi woi ak fecha lang de deff si guenteyii?*

- 1. How does it make the woman feel? (OR how did you feel at your child’s naming ceremony?)

*Ban fasong ngi yeek-yeek la de jokh gigen bi si bessi guente bi?*

1. What group-based activities do you think might help women deal with stress, anxiety, and depression if they came together regularly during pregnancy/after delivery?

*Yan fasong ngi khew-khew wii mbolo nga fok neh dina dimbaleh gegen yii sung hameh nakhare, jahleh, ak halat bu barreh su fekek nyu ngi ham dageeh wakh tu bu neeka sung birreh ak sung wosine?*

1. Do you think singing together would be beneficial for women who are experiencing stress, anxiety, or depression during and after pregnancy? If so, why? If not, why not?

*Ndakh fox nga neh woi mbolo dina ham njerign si gigen yii ham nakhare, jahleh, walla helbu dalut su nyu birree ak su nyu wosine? Su hameh, lan mo takh? Walla su hamute?*

- 1. Do you think it might be beneficial for some women and not others?

*Ndakh fox nga ne di ham njerign si yee baayi yee?*

1. What kind of singing or music do you think would be beneficial (if any)?

*Yan fasong ngi woi walla misik nga fox ne di ham njerign? (su ameh)?*

- 1. Would it be beneficial to teach pregnant mothers lullabies to sing to their babies?

*Ndakh fox nga neh di ham njerign purr jangal gigenii birr yii woi yii lirr yii purr nyu ko woiyal sen doom yii?*

**Griots**

1. Can music help people deal with their stress, worries, and concerns? If so, how? *Ndakh misik muna dembaleh nengi si sen hol bu nakhare, jahleh, ak buga-buga yii?Su deh neka na, nakala?*
2. What kinds of support do you give to people experiencing sadness or mental distress?

*Yan fasong ngi ndimbal ngai jokh nengi nyu nga ham neh nyngi ham hol bu nakhre, walla hel bu dalut?*

1. What kinds of songs are sung to babies? Follow up: Can you sing any of these songs?

*Yan woi lanyo woyal lirryi? Ndakh mun nga woi bena si woyi?*

- 1. Who knows these songs and who sings them to babies?

*Kan mo kham woyi ak kan moi ko woyal lirryi?*

- 1. What is the benefit of these songs/ why are these songs performed?

*Lan moi njerign woi ak fechayi/ lan motakh nyun koi deff?*

1. What kinds of songs and dances are performed at naming ceremonies?

*Yan fasong ngi woi ak fecha lang de deff si guenteyii?*

- 1. What is the benefit of these songs and dances/why are they performed?

*Yan fasong nginjerign la woi ak fecha yii ham/ ak lan motakh nyu koi deff?*

- 1. How does it make the woman feel? (OR how did you feel at your child’s naming ceremony?)

*Ban fasong ngi yeek-yeek la de jokh gigen bi si bessi guente bi?*

1. What group-based activities do you think might help women deal with stress, anxiety, and depression if they came together regularly during pregnancy/after delivery?

*Yan fasong ngi khew-khew wii mbolo nga fok neh dina dimbaleh gegen yii sung hameh nakhare, jahleh, ak halat bu barreh su fekek nyu ngi ham dageeh wakh tu bu neeka sung birreh ak sung wosine?*

1. Do you think singing together would be beneficial for women who are experiencing stress, anxiety, or depression during and after pregnancy? If so, why? If not, why not?

*Ndakh fox nga neh woi mbolo dina ham njerign si gigen yii ham nakhare, jahleh, walla helbu dalut su nyu birree ak su nyu wosine? Su hameh, lan mo takh? Walla su hamute lan mo ko waral?*

- 1. Do you think it might be beneficial for some women and not others?

*Ndakh fox nga ne di ham njerign si yee baayi yee?*

1. What kind of singing or music do you think would be beneficial (if any)?

*Yan fasong ngi woi walla misik nga fox ne di ham njerign? (su ameh)?*

1. Would it be beneficial to teach pregnant mothers lullabies to sing to their babies?

*Ndakh fox nga neh di ham njerign purr jangal gigenii birr yii woi yii lirr yii purr nyu ko woiyal sen doom yii?*

**CBCs**

1. What kinds of challenges do women encounter during pregnancy and after delivery?

*Ban fasong ngi jeffe-jeffe walla tekha-tekha la gigenyi de am sunyu biree ak sunyu wosine?*

- 1. Follow up questions – what causes this challenge? What are the results/ why is it a problem?

*Lan moi waral tekha-tekha yii? Lu moi jurrel lam/ Lan mo takh mu neeka tekha-tekha?*

1. What kinds of support do CBCs offer to women during and after pregnancy? Follow up on each point.

*Yan fasong ngi ndimbal la forrkati doom di ndimbaleh gigen yii su nyu biree ak su nyu wosine?*

1. Do women sometimes experience mental health issues (e.g. sadness, worry, fear) during pregnancy or after they have given birth?

*Ndakh gigen ngi dang deh lego-leag ham hel bu dalut (si missal, nakhare, jahleh, ak ragal) su nyu biree walla su nyu hameh doom?*

- 1. (If so) Is there a special name for this?

*(su hameh) ndakh ham turee bopam?*

- 1. (If so) What are the causes of mental health issues during and after pregnancy?

*(su hameh) lan moi waral hel bu dalut si gigen bu birr ak su wosine?*

1. Is there any way to avoid experiencing mental health issues during and after pregnancy?

*Ndakh hamna hai yon yoo hamneh mon nga ko moitu si waali hel bu dalut si gigen bu birr ak su wosine?*

1. Is there any treatment for mental health issues during and after pregnancy? If so, how effective is this treatment thought to be?

*Ndakh hamna hai fasong ngi fach purr hel bu dalut si gigen bu birr ak su wosine? Su hameh, naka nga fox neh la ameh njerign?*

1. What kinds of things do women typically do to help themselves if they experience anxiety, stress, or depression?

*Yan mbirr la gigen yii warr di deff purr ndimbaleh sen bopa su nyu hameh jahleh, nakhare, walla hel bu dalut?*

1. What are the signs of mental health issues during and after pregnancy?

*Lan moi mandarga hel bu dalut si gigen bu biir ak su wosine?*

1. What are the possible effects of mental health issues during and after pregnancy? How severe can it be? (for the mother and her baby)

*Lan moi jurel lu hel bu dalut si gigen bu birr ak su wosine? Nakala bun a garawee? (si yaai bi ak doomam)*

1. What kinds of support do CBCs give to women with mental health issues during pregnancy and after delivery?

*Yan fasong ndimbal lai forrkati doom yii di joh gigen bu helam bi dalut su biiree ak su wosine?*

1. Are you involved in singing or music activities? If so, of what kind?

*Ndakh boka nga si walu yengu-yengu woi ak fecha? Su amee, ban fasong?*

1. What kinds of songs are sung to babies? Follow up: Can you sing any of these songs?

*Yan woi lanyo woyal lirryi? Ndakh mun nga woi bena si woyii?*

- 1. Who knows these songs and who sings them to babies?

*Kan mo kham woyi ak kan moi ko woyal lirryi?*

- 1. What is the benefit of these songs/ why are these songs performed?

*Lan moi njerign nyi woiyi/ lan mo takh nyu woi woyi?*

1. What group-based activities do you think might help women deal with stress, anxiety, and depression if they came together regularly during pregnancy/after delivery?

*Yan fasong ngi khew-khew wee mbolo nga fok neh dina dimbaleh gegen yii sung hameh nakhare, jahleh, ak halat bu barreh su fekek nyu ngi ham dageeh wakh tu bu neeka sung birreh ak sung wosine?*

1. Do you think singing together would be beneficial for women who are experiencing stress, anxiety, or depression during and after pregnancy? If so, why? If not, why not?

*Ndakh fox nga neh woi mbolo dina ham njerign si gigen yii ham nakhare, jahleh, walla helbu dalut su nyu birree ak su nyu wosine? Su hameh, lan mo takh? Walla su hamute lan mo ko waral?*

- 1. Do you think it might be beneficial for some women and not others?

*Ndakh fox nga ne di ham njerign si yee baayi yee?*

1. What kind of singing or music do you think would be beneficial (if any)?

*Yan fasong ngi woi walla misik nga fox ne di ham njerign? (su ameh)?*

- 1. Would it be beneficial to teach pregnant mothers lullabies to sing to their babies?

*Ndakh fox nga neh di ham njerign purr jangal gigenii birr yii woi yii lirr yii purr nyu ko woiyal sen doom yii?*

**Midwives**

1. What kinds of challenges do women encounter during and after pregnancy?
   1. Follow up questions – what causes this challenge? What are the results/ why is it a problem?
2. What kinds of support do women need during and after pregnancy? Why?

1. What kinds of support do midwives offer to women during and after pregnancy?
2. What do you understand about mental health issues during and after pregnancy?
   1. Unsteady mind
   2. Stress
   3. Trouble thinking, worries etc.
3. What are the signs of mental health issues during and after pregnancy?
4. What are the possible effects of mental health issues during and after pregnancy? (for the mother and her baby)
5. What kinds of support do you give to women with mental health issues during and after pregnancy?
6. Do women confide in midwives when they are experiencing mental distress? Can you provide examples?
7. What is the impact of midwives’ activities?
8. Are you involved in singing or music activities? If so, of what kind? E.g. we are interested in learning more about the songs and music associated with the perinatal period (during and after pregnancy).
9. What kinds of songs are sung to babies?
   1. Who knows these songs and who sings them to babies?
   2. What is the benefit of these songs/ why are these songs performed?
10. Do you think a singing-based intervention at the RCH clinic would be beneficial for women who are experiencing mental distress during and after pregnancy? If so, why? If not, why not?
    1. What kind of singing or music do you think would be beneficial (if any)? (e.g. teaching and singing lullabies for babies or songs to promote awareness of mental health issues during and after pregnancy)
    2. Do you think it might be beneficial for some women and not others?
