# Supplementary material for: The experience of maternal mental distress in The Gambia: A qualitative study identifying idioms of distress, perceptions of contributing factors and the supporting role of existing cultural practices
Source: PLOS Glob Public Health. 2023 Sep 7;3(9):e0002329. doi: 10.1371/journal.pgph.0002329 (PMC10484451; doi:10.1371/journal.pgph.0002329)
Supplement: S2 Text — (DOCX) [file pgph.0002329.s003.docx]

**S2 Text**

One Kanyeleng woman explained how feeling nervous and overthinking can contribute to someone experiencing *hel bu dalut* or *sondomoo tenkung baliyaa.*

“Anxiety/nervousness (kijafaro) can cause mental distress (sondomoo tenkung baliyaa). If you are nervous or panicky that can cause your thoughts to be many (miraalisiyaa).“ – Mandinka Kanyeleng women from Sanyang

The anxiety/nervousness described can also cause a confused or worried mind as reported by a Mandinka CBC from Farato.

“Here I am by myself without anybody, I will keep on thinking. I won’t have a stopping point. That can become a mental thinking illness (mirrakuurgo). Anxiety/nervousness (kijafaroo) is part of it.” – Mandinka CBC from Farato

Similar terms that describe overthinking, nervousness and anxiety were also found in Wolof.

“After the naming ceremony, the woman also must endeavour to come out to the people outside to chat with them. Because solitude causes many thoughts/worries (xalat bu bari)” – Wolof TC from Kissimajaw

“I don’t experience any anxiety/fearfulness (tiitangeh) or any problem. I thank God.” – Wolof pregnant woman from Faajakunda.

While panicking and worry were discussed as causes of mental distress during the perinatal period so were feelings of sadness, sorrow or misery in both Mandinka and Wolof.

“A problem starts in the compound. That is sadness (niikuyaa) and distress/misery (niitooroo). […] You are sitting and the husband should give you happiness/a peaceful soul (niidiyaa) but you don’t get happiness from him” – Mandinka Kanyeleng from Sanyang

“Being in a matrimonial house and yet the husband squanders his wealth away somewhere is enough reason to have sorrow (Naxarr).” – Wolof CBC from Kissamajaw

These quotes give a variety of helpful terms to describe several different relevant emotions such as, sadness (niikuyaa in Mandinka or Naxarr in Wolof), misery (niitooroo), and happiness (niidiyaa or niilaa).
