# Supplementary material for: The experience of maternal mental distress in The Gambia: A qualitative study identifying idioms of distress, perceptions of contributing factors and the supporting role of existing cultural practices
Source: PLOS Glob Public Health. 2023 Sep 7;3(9):e0002329. doi: 10.1371/journal.pgph.0002329 (PMC10484451; doi:10.1371/journal.pgph.0002329)
Supplement: Attachment — Submitted filename: Response to reviewers_FINAL.docx [file pgph.0002329.s004.docx]

We would like to thank the editor and all reviewers for their thoughtful and helpful comments. Below we detail our responses to each reviewer’s comment (in italics) as well as highlight any substantial changes by including the new text in this document (with page numbers). In the attached revised manuscript, we have used track changes to highlight all substantial and minor changes. We have also taken the opportunity to make some small re-wording edits throughout to improve flow and readability.

**Editor Comments:**

1. *This manuscript is very well-written and offers important insights into cultural manifestations of maternal mental health. The edits recommended by the reviewers will improve the clarity and potential for impact of the paper.*

Thank you for your helpful comments.

2. *I agree with the reviewer's suggestion to provide additional information on the participant observations in the methods section. See the COREQ guidelines for additional suggestions of information to add to the methods. It would also be helpful to provide the FGD guide(s) and final codebook as supplemental materials.*

We have now added more information throughout and provided the COREQ checklist and FGD guides as supplementary material.

1. *Although the strengths of the approach are provided in the discussion, it should be paired with a description of the limitations of the study.*

A ‘Limitations and future directions’ section is now included.

2. *Please also consider the recommendations related to the Social Context theme.*

We have taken on reviewer 2’s recommendations and split this theme into two separate themes: (1) Poverty of the healthcare system and (2) Shifting cultural context.

3. *One of the reviewers provided additional detailed feedback in a pdf--please reach out if you cannot access or view this document.*

These have been integrated into the changes in the document but not explicitly responded to in this letter. These changes can be seen throughout in the track changes. The most substantial changes were adding more detail into the methods section, splitting ‘Societal Context’ into two themes, and renaming the final theme to ‘Cultural practices involving music’.

**Journal Requirements:**

1. *Please review your reference list to ensure that it is complete and correct. If you have cited papers that have been retracted, please include the rationale for doing so in the manuscript text or remove these references and replace them with relevant current references. Any changes to the reference list should be mentioned in the rebuttal letter that accompanies your revised manuscript. If you need to cite a retracted article, indicate the article’s retracted status in the References list and also include a citation and full reference for the retraction notice*.

We have checked the reference list and found no papers that have been retracted. We have added two new references:

One reference is a recent related paper which we have published:

Sanfilippo KRM, Glover V, Cornelius V, et al. Expression of antenatal symptoms of common mental disorders in The Gambia and the UK: a cross-sectional comparison study. BMJ Open 2023;13:e066807. doi: 10.1136/bmjopen-2022-066807

The other is cited to avoid naturalising poverty by referencing this work on how it is produced and the impact on maternal health.

Simona, Simona. "Structural violence and maternal health care in sub-saharan africa: A theoretical perspective." Social Sciences 11.4 (2022): 194-200. doi: 10.11648/j.ss.20221104.11

2. *Please amend your detailed Financial Disclosure statement. This is published with the article. It must therefore be completed in full sentences and contain the exact wording you wish to be published.*
   a. *State what role the funders took in the study. If the funders had no role in your study, please state: “The funders had no role in study design, data collection and analysis, decision to publish, or preparation of the manuscript.”*
   b. *. If any authors received a salary from any of your funders, please state which authors and which funders.*
   c. If you did not receive any funding for this study, please simply state: “The authors received no specific funding for this work.”

We have now amended this statement and added information about authors who received salary.

“The funders had no role in study design, data collection and analysis, decision to publish, or preparation of the manuscript. KRMS and BM’s time and travel expenses were covered as a part of this project’s funding. BD was paid for his time on the project. LS had her time bought out and HBH was paid as a full-time research assistant on this project.”

**Reviewer #1:**

1. *This is a well-written paper which explores local conceptualisations of perinatal distress, as well as the economic and sociocultural factors which can impact on perinatal mental health and wellbeing. The study is well-conducted and will be an important contribution to the field. Below are some comments/suggestions, which should be considered prior to publication.*

Thank you for your helpful comments and feedback.

2. *Lines 83-87 it is stated that “Moreover, specific social factors in various LMICs present additional risks to maternal mental health. For example, a study by Wittkowski et al. (15) identified four main factors contributing to postnatal depression symptoms in Sub-Saharan Africa: lack of social support (especially from the husband), relationship problems, an unwanted pregnancy, and cultural factors, such as family structures or polygamy.” – it might be argued that (apart from polygamy) that these are universal predictors of poor maternal mental health, not just specific to LMICs; therefore perhaps be more tentative with this statement.*

We appreciate this feedback. We have revised that section to be clearer and deleted the first sentence.

“In the sub-Saharan African context, a study by Wittkowski et al. (13) identified four main factors…” (Introduction, Page 3, Line 85)

Also, based on suggestions in the PDF from reviewer 2 we have made more explicit throughout which context we are discussing when summarising previous literature. (See track changes throughout document)

3. *Line 126: I recommend that “griot” is defined briefly here for those not familiar with the term*

The text already includes a definition for ‘griot’. We have revised the wording slightly to provide more clarity.

“These cultural practices are led by specialists such as griots, defined as hereditary musical experts and knowledge holders who play important cultural roles as communicators, emotional transformers and conflict mediators (17)..” (Introduction, Page 5, Line 135 -137)

4. *Lines 177-178: it is noted that the research team engaged in participant observation to gain an understanding of cultural ceremonies in the perinatal period. Are these observations included as part of the analysis in the current paper? If so, more information about these observations should be included (e.g. which ceremonies, frequency etc).*

We have added additional information about this aspect of the research. Field notes were not included in the analysis. The field work was used as a way for the research team to have a better contextual knowledge of Kanyeleng practices. We have therefore moved this information to the beginning of the methodological approach section.

“The research team (KRMS, BM, BD, HBH, MG) engaged in participant observation (2018-2019) to gain an understanding of tacit knowledge relating to cultural ceremonies during the perinatal period. Participant observation involved regular attendance and participation in naming ceremonies (kunliyoolu, Mandinka) and Kanyeleng performances of various kinds, such as initiation ceremonies for new Kanyeleng (kuroo), and special Kanyeleng naming ceremonies for children born to Kanyeleng women. While field notes were not included in the analysis, participant observation provided the necessary contextual knowledge through which to understand the examples and concepts discussed in the focus group discussions ” (Methodological approach, Page 7-8, Lines 262 -270)

5. *Line 188 – how are research sites defined (e.g. geographical location/setting?)*

Research sites were defined based on geographic location. We have added more clarity to how these were defined.

“Research sites were defined based on geographic location and were selected, through consultation with our Ministry and NCAC partners, to include participants from two major language groups, Mandinka and Wolof. “(Setting and participants, Page 9, Lines 321 - 324)

6. *There is no information about how the different participants were recruited/identified, and the procedures involved. I recommend that the authors looks at guidelines for the reporting if qualitative studies (e.g. COnsolidated criteria for REporting Qualitative research (COREQ) checklist; Tong et al., 2007) to ensure important information is reported in the manuscript (e.g. relationship of interviewees to participants etc). A copy of the topic guide should also be included (i.e. as supplementary file)*

We have now included more information throughout the methods section and completed the COREQ check list which is now attached as Supplementary Material 1 along with the FGD topic guides (Supplementary Material 2). The more substantial changes are seen below:

“The Consolidated Criteria for reporting qualitative research (COREQ) (31) was used and the COREQ checklist can be found in Supplementary Material 1.” (Materials and methods, Page 6, Lines 160-162)

“BM (female), an ethnomusicologist, and BD (male), head of health communication at the Ministry of Health, have extensive expertise running focus groups in The Gambia. BM trained the research assistants (RAs) (Hajara B Huma (HBH) (female) and Malick Gaye (MG) (male)) in running semi-structured FGDs. Both HBH and MG are trained psychiatric nurses working in The Gambia. At least two members of the research team who spoke Mandinka or Wolof (BM, BD, HBH, MG) were present at each focus group discussion. BD, BM both have extensive experience working with Kanyeleng groups and therefore had some professional relationships with the participants. KRMS (female) spent time working with colleagues in The Gambia but did not run the FGDs as she does not speak either local language. (Study team, Page 7, lines 251-259)

“CBCs, Midwives, and Kanyeleng/TC groups were all invited by phone and in person to participate by our Ministry of Health contact (Buba Darboe). Musicians and griots were invited by phone and in person to participate by our NCAC contact (Hassoum Ceesay). Pregnant women were invited to participate in person by nurses and midwives during their reproductive and child health clinic visits across three clinics (Table 1)”. (Setting and participants, Page 10, Lines 343-347)

7. *There was very little information regarding the demographic characteristics of participants, especially the pregnant women (e.g. age, parity, marital status, employment), which are likely to have an impact on their experiences of pregnancy and motherhood. If these have been collected, it would be helpful if these can be summarised in the manuscript. Similarly, with other participants it might be helpful to know some of their demographic (e.g. age/gender) and professional (where appropriate) characteristics (e.g. how long have they been a midwife).*

We did not collect detailed demographic characteristics of the participants in the focus groups. The focus group discussions took place in group settings with little privacy due to the crowded nature of the clinics. It was not considered appropriate by our local partners to ask personal demographic questions of participants in this context. The research team focused on developing rapport and a comfortable environment to encourage participants to engage in discussion. However, another study we conducted did collect more detailed demographic information on pregnant participants attending the same antenatal clinics. We have therefore added that this detail on general demographics of pregnant women attending antenatal clinics in The Gambia can be found in another piece of work we completed.

“No additional demographic characteristics were collected. However, a previous study conducted by the same research team collected more detail demographic data about the pregnant women attending the same antenatal clinics. More information about the pregnant women who attend these clinics can be found in Sanfilippo et al., (32)” (Setting and participants, Page 11, Lines 352 - 355)

8. *Lines 239-241: were there any differences in the conceptualisations of distress across the different FGDs or was this the general consensus? I think the explanation around stigma and the different idioms used to describe distress could be expanded on – did these perceptions of stigma come from the different groups, or from the research team?*

Conceptualisations of distress and stigma are highly complex and varied between individuals and between different specialist groups. Our understanding of the idioms of distress is based on the terms used across all the focus group discussions conducted in the Mandinka and Wolof languages (the midwives FGDs were conducted in English and therefore did not inform our understanding of local language idioms of distress). In addition, we engaged in extensive consultation within the research team (which included two experienced mental health nurses) and with language experts at the National Centre for Arts and Culture. While the idioms of distress deserve more extensive, in-depth research and analysis, as they shape the way that perinatal mental distress is experienced and understood, this is beyond the scope of the current project.

In our ‘Results and Discussion’ section we added:

“Part of our analytical approach was to ensure that local terms were noted throughout the analysis procedure. This was to help inform the analysis of this work as well as to inform the development of a translated tool used to measure symptoms of maternal distress (32). Conceptualisations of distress and stigma were highly complex and varied between individuals and between different specialist groups. A constellation of terms and phrases (idioms of distress) were identified to describe some of the emotional states a woman can experience in pregnancy and after birth. Our understanding of these idioms of distress is based on the terms used across all the focus group discussions conducted in the Mandinka and Wolof languages (the midwives FGDs were conducted in English and therefore did not inform our understanding of local language idioms of distress).” (Results and discussion, Page 13, Lines 399-407)

In our ‘Limitations and future directions’ section we added:

“Our approach to identifying idioms of distress was based on extensive consultation within the research team (which included two experienced mental health nurses) and with language experts at the NCAC. However, the identification and understanding of the idioms of distress deserves more extensive, in-depth research and analysis, as they shape the way that perinatal mental distress is experienced and understood. However, this was beyond the scope of the current project.” (Limitations and future directions, Page 34, Lines 1006 - 1010)

9. *I am not convinced that “Social Context” necessitates a distinct theme from the other themes i.e. Economic Factors, Social Factors, and Spiritual Factors, as the wider social context intersects with, and is important within, these three themes (e.g. pregnancy out of wedlock is discussed in social context but is also a social factor). Please consider whether a separate theme is needed here, or if the theme needs further refinement/development (e.g. healthcare systems seems important and distinct from the others).*

We have taken on Reviewer 2’s recommendation around this theme and split this theme into two separate themes: (1) Poverty of the healthcare system and (2) Shifting cultural context.

10. *Lines 324-327: it is not clear how these cultural shifts in values can negatively (or positively) impact women and/or mothers – can this argument be developed further?*

We have added further explanation of this point.

Some viewed these changes as positive, leading to increased freedom and opportunities for women. Other participants viewed them as causing more financial and mental strain on mothers as gender roles and behaviours are renegotiated, potentially leading to conflict within the family and increased responsibilities for women both within and outside the home. (Shifting cultural context, Page 18, Lines 579 - 583)

11. *I would have expected to see a discussion/recognition of the strengths and limitations of the research. How were quality and trustworthiness ensured in this study?*

We have added a subsection in the Conclusions section titles ‘Limitations and future directions’

Our research sought to gain an understanding of participants lived experiences and perspectives through qualitative methods of focus group discussions and participant observation. We sought to ensure the quality and trustworthiness of the research by 1) including multiple perspectives (i.e. conducting focus groups with different knowledge holders in order to avoid privileging the perspective of a particular group), 2) engaging in participant observation to gain contextual understanding to aid in the interpretation of themes identified in the focus group discussions, and 3) working collaboratively as an interdisciplinary and intercultural research team to ensure that biases based on disciplinary or cultural perspective did not shape our interpretation of the results. This is described in the ‘Methodological approach’ section of the paper.

“The methodological approach was designed to engage marginalised perspectives that have not been captured in existing research on women’s mental health in West Africa, despite their local cultural significance and influence. It employed a collaborative approach, through our partnership with the Ministry of Health and the National Centre for Arts and Culture, that aimed to enable the research to be enriched by interdisciplinary research expertise while remaining grounded in the priorities of Gambian partner organisations and research participants. The interdisciplinary approach aimed to take local knowledge systems, held by local women’s groups like the Kanyeleng, as the starting point for building an understanding of women’s experiences during the perinatal period. Participant groups were selected to incorporate cultural expertise as well as knowledge of women’s health in the local context.” (Methodological approach, Page 8, Lines, 278-287)

12. *What implications for future research does this project have? What are the recommendations for researchers working in this field?*

We have added a subsection in the Conclusions section titled ‘Limitations and future directions’. We end with some general discission of recommendations for researchers in the field:

“We argue that developing a fuller understanding of these local perspectives on experiences of perinatal mental distress, contributing factors and coping strategies is necessary to inform culturally relevant global health initiatives that align with local priorities (e.g., (42,51)). This is important in moving beyond outsider-driven responses that fail to adequately engage with local knowledge systems and culture. This may be particularly necessary when addressing perinatal mental health, a neglected women’s health issue subject to widespread stigma.” (Conclusions, Page 35, lines 1037 – 1043)

13. *Ensure statements in the discussion are grounded in the data. For example, “hiding pregnancies” (Line 659), is not described in the results.*

This has now been taken out of the discussion and we have ensured all discussion of the results is grounded in the data.

**Reviewer #2:**

1. *I find this a very interesting and relevant study that is very worth publishing, but I have some comments and suggestions that I have made on the PDF itself which would contribute towards increased scientific rigour and reliability of your reporting and of the article itself. Once all of these comments have been addressed I would be very happy to see this paper published. I do not necessarily need to see your responses to these comments.*

Thank you for your comments and the more detailed comments in the attached PDF. We have made changes in relation to the attached comments. These changes can be seen in the track changes. The most substantial changes were to add more detail into the methods section, splitting ‘Societal Context’ into two themes, and renaming the final theme to ‘Cultural Practices Involving Music’.

2. *Page7:
   I would be interested to know how you got to identifying the idioms of distress when it doesn’t appear to have been a particular theme or line of questioning in your focus group interview schedules (unless I missed it).*

The idioms of distress were not explicitly asked about in the focus group discussions, but we identified this as an important aspect during the analysis. That is, the discussions revealed important insights into the way experiences of mental distress were described in the Mandinka and Wolof languages. We have added more clarity in how and why this was done in the Results and Discussion section and the Limitations and Future Directions section. (See Reviewer 1 point 8)

3. *Page 8: you make no mention of any results from your participant observation methods – please either incorporate this throughout or remove this as a method.*

We have added more clarity to this point. See our response to Reviewer 1 point 4.

4. *Page11: results:
   I have a query about the structure of your results – I have not come across the way you have presented the results before in mental health literature – i.e. incorporating external data and literature in your results. This may be acceptable by the journal but I would then tend towards labelling this section as ‘results and discussion’ rather than just results, and then move some of your discussion up into the current ‘result’s section, and then add a concluding section to sum up your findings and recommendations for future research, practice, and policy.*

We appreciate this suggestion. The presentation of results and references to other studies reflects the interdisciplinary composition of the research team, which includes scholars from humanities as well as health science disciplines. Norms of research writing across these different fields vary, but we have sought to present the findings in a way that will be accessible to a wide audience and provide a clear understanding of this complex topic. In certain places we felt this required inclusion of references to other scholarship alongside the presentation of our results (this is common in humanities and some social science writing).

This interdisciplinary is briefly addressed in the ‘Methodological approach’ section. For more clarity we have also decided to rename the “Results” section “Results and Discussion” and then change the “Discussion and Conclusions” to just “Conclusions” to reflect the type of material included in each section. This type of structure of reporting is common in the humanities disciplines reflected in the research team.

5. *Page 24
   I have made a few substantial comments about the theme ‘cultural practices’ and its singular orientation towards music.*

We have made some edits in response to this feedback about the representation of this theme. We have renamed the section “Cultural Practices Involving Music”. We have also changed the word “identified” to “described” in the first sentence of the section. We have also made some other small edits throughout this section and in the discussion to be clear about this theme’s focus on music.

6. *Discussion and conclusion: I would also like to see any limitations of the study mentioned. (e.g. the fact that the data is quite a few years old)*

We have added a subsection in the conclusions section titled ‘Limitations and future directions’. While the data is from 2018, we do feel that it is still relevant to women’s current experience so do not see this as a limitation of the work.

**“Limitations and future directions**

Our approach to identifying idioms of distress was based on extensive consultation within the research team (which included two experienced mental health nurses) and with language experts at the NCAC. However, the identification and understanding of the idioms of distress deserves more extensive, in-depth research and analysis, as they shape the way that perinatal mental distress is experienced and understood. However, this was beyond the scope of the current project.

Some participants might have feared disclosing their own experiences of mental health within a focus group environment, especially as mental health problems are stigmatised in The Gambia. To help reassure participants, the facilitators instructed participants that information disclosed in the discussion should not be shared outside the group during the consenting process. Participants were also invited to talk about experiences they have heard from others rather than their own personal experiences.

Most FGDs were held at health centres and all pregnant participants were recruited from antenatal clinics. This may have led to some participants discussing treatments and concepts believed to be valued within the biomedical model of care. In addition, the sample of pregnant women was using the clinics. Therefore, we were not able to sample perceptions from women who were not seeking or could not seek care from the antenatal clinics in the first place. Future research could directly target women who do not use the clinics to better understand how their experiences might be similar or different to those discussed within this current work.**”** (Page 34, Lines 1005 – 1025)

**Reviewer #3:**

1. *Thank you for sending this paper - I thoroughly enjoyed reading it, and recommend for publication. This is a beautifully researched and written paper which highlights very clearly a vital issue in maternal mental health, the importance of understanding the cultural context. I struggle to find issues to revise for review, to be honest, and can recommend this paper as it is for publication. The following are my reflection on this article, rather than issues for correction*

Thank you for your kind words!

2. *For myself I would have been interested in further elaboration of the linguistic issues, the idioms used and why each term was more or less stigmatising.*

See response to Reviewer 1 (point 8) above. We have also added that we feel this should be explored in more depth in a future research study and have therefore added this point to the new ‘Limitations and future directions’ section

“Our approach to identifying idioms of distress was based on extensive consultation within the research team (which included two experienced mental health nurses) and with language experts at the NCAC. However, the identification and understanding of the idioms of distress deserves more extensive, in-depth research and analysis, as they shape the way that perinatal mental distress is experienced and understood. However, this was beyond the scope of the current project.” (Limitations and future directions, Page 34, Lines 1006 - 1010)

3. *I would also have been interested in further description, and perhaps a stronger stress upon, the power relations inherent in the family relationships within homes/compounds. The difficulty for (especially young) women in being given permission to attend medical appointments is something I remember well, and saw starkly for those who had tested HIV+ in pregnancy. For many women and girls to manage to attend appointments takes a persistence and sometimes bravery that should not be understated.*

This is an important point. We have added an additional sentence on this topic:

“Conflicts within the family emerge in the context of a gendered division of labour and women’s extensive domestic responsibilities. Unequal power relations within the family mean that individual women may not have the freedom to make their own decisions about maternal health care and must receive permission from their husbands or in-laws to attend clinic appointments.” (Social factors, Page 20, Lines 632 – 635)

4. *While I agree and appreciate that music is key to understanding the ways in which cultural values are transmitted, and support and community offered to women through these, I also felt that the importance of food in these ceremonies and in the cohesion of social life could also come through more strongly. There are important and insightful mentions throughout of the ways in which food plays into these stresses and supports, and these could be tied together in the discussion, in a similar way to the theme of music and culture. Sitting together and eating together are demonstrations of community - and being alone or isolated is more than just stressful. In my research people talked about the isolation of HIV stigma - and specifically eating alone - being as deadly as the disease itself.*

This is a great point and something we saw in our research as well. While we feel we cannot go into this in depth within this paper we have added mention to this within the ‘Cultural Practices Involving Music’ section

“Additionally, engagement with musical practices was often described as closely connected to other aspects of culture, such as food. Kanyeleng ritual is a site where song, dance, food, comedy, and prayer are combined with the goal of promoting social and spiritual health.” (Cultural practices involving music, Page 28-29, Lines 249 – 245)

5. *Similarly the performative nature of clothing and ceremonies - being able to host a ceremony and have new clothes, could be made clearer - although this sits within the economic factors section there are broader societal norms at play here as well, that may not be clear to many readers.*

We have added a sentence to provide a clearer indication of the significance of clothing in the context of naming ceremonies:

“Along with music and food, having new clothes to wear during a naming ceremony is part of the performative celebration of a new mother, reflecting the broader social significance of clothes in notions of femininity and women’s social networks in the Gambia.” (Economic factors, Page 22, Lines 687 – 690)

6. I also wondered about the degree to which this project was able to make comment on those women and girls who were more or less able or likely to access care - I appreciate this may be out of scope for this research but would be interesting to understand further.

We agree and feel that this is something that could be an interesting future piece of work and have added this to the ‘Limitations and Future Directions’ section.

Most FGDs were held at health centres and all pregnant participants were recruited from antenatal clinics. This may have led to some participants discussing treatments and concepts believed to be valued within the biomedical model of care. In addition, the sample of pregnant women was using the clinics. Therefore, we were not able to sample perceptions from women who were not seeking or could not seek care from the antenatal clinics in the first place. Future research could directly target women who do not use the clinics to better understand how their experiences might be similar or different to those discussed within this current work.**”** (Limitations and future directions, Page 34, Lines 1019 – 1025)
